# Genome‐Wide Meta‐Analysis of Parkinson's Disease Associated Genetic Loci and Validation of Therapeutic Targets

**DOI:** 10.1002/brb3.71087

**Published:** 2025-11-17

**Authors:** Shan Yang, Qinfen Wu, Xinling Yang

**Affiliations:** ^1^ Xinjiang Key Laboratory of Neurological Disorder Research Urumqi China; ^2^ Department of Neurology The Second Affiliated Hospital of Xinjiang Medical University Urumqi Xinjiang China

**Keywords:** Parkinson's disease, genome‐wide meta‐analysis, therapeutic genes, statistical methods, drug prediction

## Abstract

**Background:**

To identify genetic loci associated with Parkinson's disease (PD) through a genome‐wide meta‐analysis, to screen for druggable genes significantly linked to PD risk, and evaluate their potential as therapeutic targets.

**Methods:**

Data from DGIdb, GeneCards, and the Finan genomic resource were integrated to identify candidate therapeutic genes associated with PD. Genome‐wide meta‐analysis was conducted using GWAS data from the International Parkinson's Disease Genomics Consortium and FinnGen, involving 1,851,374 participants. Mendelian randomization (MR), colocalization analysis, and phenome wide association studies (PheWAS) were conducted to validate the associations between the identified genes and PD. Furthermore, knockout mouse models from the Mouse Genome Informatics were analyzed to validate PD‐related phenotypes, and DSigDB was utilized to predict potential therapeutic compounds.

**Results:**

We identified several therapeutic genes significantly associated with PD risk. Colocalization analysis suggested shared causal genetic variants between these genes and PD. PheWAS further revealed that GCLC and GFPT1 exhibit limited pleiotropic effects across other traits. Eight potential compounds were identified through DSigDB predictions.

**Conclusion:**

Through genome‐wide meta‐analysis, MR, colocalization, and PheWAS, we identified genetic loci associated with PD and assessed GCLC and GFPT1 as potential therapeutic targets.

## Graphical abstract

This study identified genetic loci associated with Parkinson's disease through a genome‐wide meta‐analysis, screened druggable genes significantly linked to PD risk, and assessed the potential of GCLC and GFPT1 as therapeutic targets.

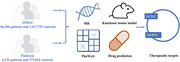

## 1 Introduction

Parkinson's disease (PD) is a common neurodegenerative disorder that primarily affects the elderly (Min et al. 2022). Its primary pathological feature is the degeneration of dopaminergic neurons in the substantia nigra (Miranda et al. 2025), leading to impaired motor control (Ryman and Poston 2020). By 2050, the number of individuals with PD worldwide is expected to reach 25.2 million, a 112% increase from 2021. Approximately 89% of this growth is attributed to population aging (Su et al. 2025). Approximately one‐third of patients exhibit mild cognitive impairment at initial diagnosis, including deficits in attention (Fan et al. 2020), executive function (Turner et al. 2021), language (Liu et al. 2015), memory (MacDonald et al. 2019), and perception (Jaywant et al. 2016). Typical symptoms of PD include postural instability, muscle rigidity, resting tremor, and bradykinesia (Chu et al. 2024; Eisinger et al. 2017; Dietrichs et al. 2023). As the disease progresses, voluntary motor control deteriorates, often accompanied by dysphagia, speech impairment, and gait disturbances (Suttrup and Warnecke 2016; Ramig et al. 2018). The absence of reliable early screening methods often leads to misdiagnosis in elderly patients. Globally, over 10 million people are affected by PD, and its incidence is expected to rise with an aging population (Li et al. 2025).

Current treatments for PD mainly rely on pharmacological interventions, including levodopa (L‐DOPA) and dopamine receptor agonists (DRAs) (Pirker et al. 2023). These drugs help restore dopamine levels and alleviate motor symptoms. L‐DOPA, a dopamine precursor capable of crossing the blood–brain barrier, remains a cornerstone of PD therapy (Cao et al. 2020). Despite therapeutic and diagnostic advances, no current treatment halts disease progression or achieves full reversal. Genomic advances have enabled the identification of numerous genetic loci associated with PD (Ryu et al. 2020). The genetic architecture of PD is complex, involving multiple genes and environmental factors (Bellou et al. 2016). Current methodologies face limitations in fully elucidating these interactions. Genome‐wide meta‐analysis has become a pivotal tool in PD genetics, integrating data from multiple studies to generate novel insights (Oh et al. 2023). A recent study integrated data from the GTEx project and three large‐scale PD genome‐wide association studies (GWAS) to identify 37 conserved imputed gene signatures from whole blood using a random forest classifier (Chew et al. 2024). These gene signatures successfully differentiated PD patients from healthy individuals, supporting their potential as novel whole‐blood biomarkers for PD. A GWAS of 28,568 PD patients revealed strong associations between age at onset and established risk loci, including SNCA and TMEM175 (Blauwendraat et al. 2019). Although over 90 PD risk loci have been identified by GWAS, their functional interpretation and translation into therapeutic targets remain major challenges (Nalls et al. 2019). Approximately 98% of GWAS signals lie in non‐coding regions, and the link between their regulatory mechanisms and druggability remains poorly understood. To uncover novel traits, we employed phenome wide association studies (PheWAS) (Bastarache et al. 2022), a hypothesis‐free approach for exploring genetic causality. This method begins with specific genetic variants and systematically investigates a broad range of human phenotypes. PheWAS is particularly effective in identifying pleiotropy and elucidating how single genes influence multiple traits (Li et al. 2021).

In this study, we integrated GWAS data from the International Parkinson's Disease Genomics Consortium (IPDGC) and FinnGen to conduct large‐scale genomic analyses. Our goal was to identify loci significantly associated with PD risk and prioritize potential therapeutic target genes. We combined Mendelian randomization (MR), colocalization, and PheWAS to validate the functional relevance of these genes and evaluate their therapeutic potential.

## 2 Materials and Methods

### 2.1 Therapeutic Genes

We integrated multiple data sources to identify potential therapeutic targets for PD. The drug–gene interaction database (DGIdb, https://www.dgidb.org/) contains over 14,000 drug–gene interactions involving 2600 genes and 6300 drugs, offering both known and predicted gene–drug associations to support drug development (Cannon et al. 2024). Additionally, we incorporated data from Finan et al., who expanded the therapeutic gene set by analyzing disease‐associated single nucleotide polymorphisms (SNPs) from GWAS, extending the list of approved targets since 2005 (Finan et al. 2017). We retrieved PD‐related genes from the GeneCards database (https://www.genecards.org/) using the keyword “Parkinson's disease” and applying a relevance score threshold >1 to generate a high‐confidence PD gene set (Fishilevich et al. 2016). Genes identified in all three sources—DGIdb, Finan dataset, and GeneCards PD gene set—were selected as candidate therapeutic targets.

### 2.2 Clinical and Demographic Characteristics

**FIGURE 1:**
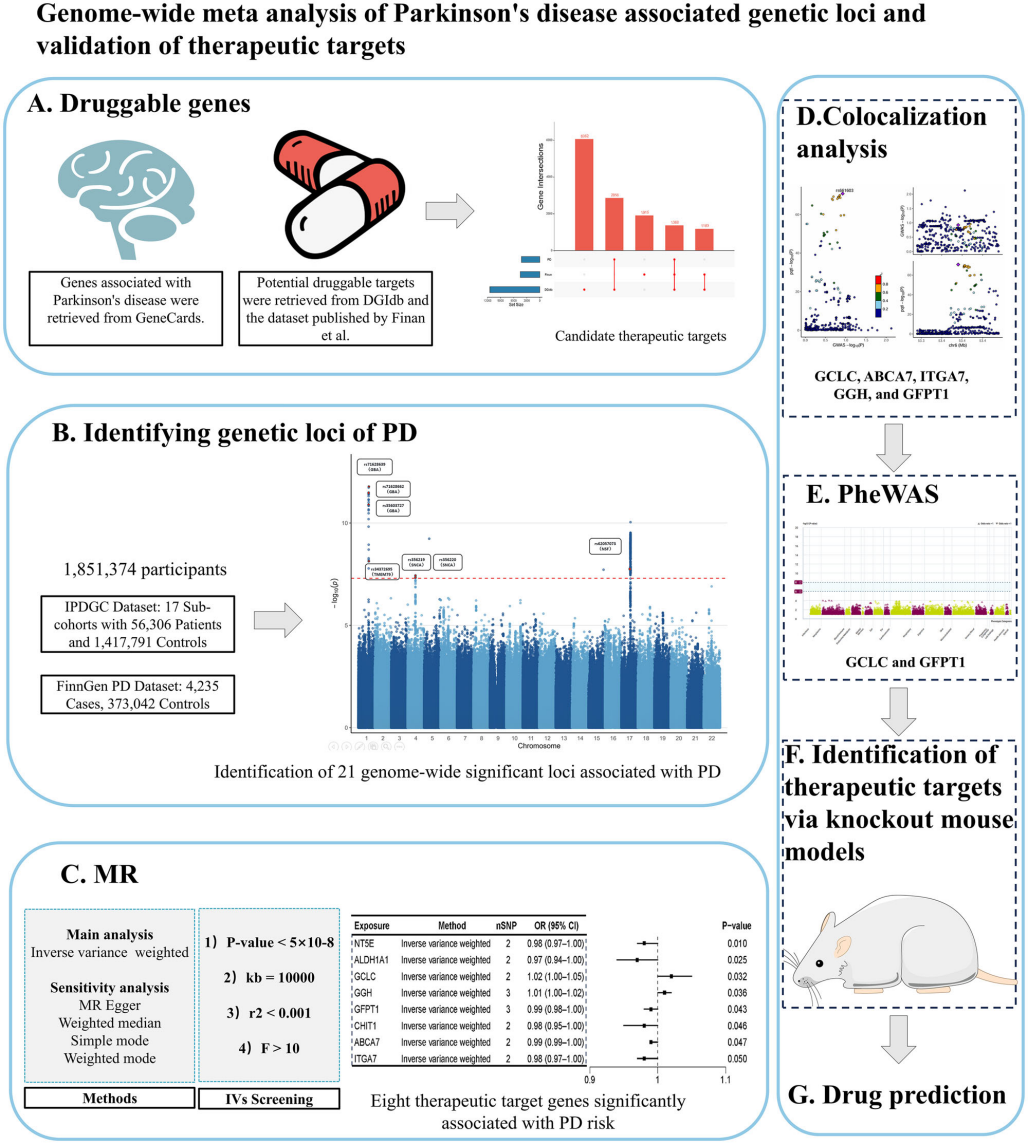
Flowchart of the study.

This meta‐analysis included data from 1,851,374 participants, sourced from two independent PD GWAS datasets. The first dataset was derived from 17 sub‐cohorts integrated by IPDGC, comprising 56,306 patients and 1,417,791 controls. The second dataset, obtained from the FinnGen R9 database, comprised 4235 cases and 373,042 controls, covering a total of 20,170,236 SNPs. Detailed clinical and demographic characteristics are available in prior publications. All study protocols received approval from the respective institutional review boards. A summary of the research process is presented in Figure 1.

### 2.3 Genome‐Wide Meta‐Analysis for Identifying Genetic Loci of PD

We conducted a meta‐analysis of summary statistics from two independent PD GWAS datasets. The data included SNP identifiers (rsid), chromosomal positions, effect alleles (EA), non‐effect alleles (NEA), effect sizes (Beta), standard errors (SE), and p‐values. Variants with >5% missing genotype rate and minor allele frequency (MAF) <1% were excluded. Meta‐analysis was performed using METAL software, with data preprocessing conducted in R version 3.6. All analyses were executed on a Linux server.

### 2.4 Gene Annotation and Enrichment Analysis

Genome‐wide significant loci (*p* < 5 × 10^−6^) were annotated using the Ensembl gene annotation framework. Significant SNPs were mapped to the nearest gene within a ±500 kb window, applying a linkage disequilibrium threshold of *r* ^2^ < 0.001. Gene symbols were standardized to ENTREZ IDs using the org.Hs.eg.db database. Pathway enrichment analysis was performed with the following parameters: minimum gene set size = 1, unadjusted *p*‐value threshold = 0.05, and false discovery rate (FDR) *q*‐value cutoff = 0.2.

### 2.5 Genetic Regulation of Therapeutic Targets in Relation to PD

eQTL data were obtained from the eQTLGen Consortium (https://eqtlgen.org/), comprising around 32,000 individuals and approximately 17,000 genes derived from peripheral blood samples of healthy European individuals (Võsa et al. 2021). SNPs were selected based on the 1000 Genomes reference panel (Byrska‐Bishop et al. 2022), using the following criteria: *p* < 5 × 10^−8^, *r* ^2^ < 0.001, and F‐statistic >10. MR analysis was performed using the TwoSampleMR package. The Wald ratio method was applied for single SNP genes, while inverse variance weighting (IVW) was used for multiple instrument genes. Results were presented as odds ratios (OR), quantifying the relationship between gene expression and PD risk.

### 2.6 Colocalization Analysis to Validate Therapeutic Target–PD Associations

To validate the causal relationships identified through MR, we performed colocalization analysis for the genomic regions of candidate therapeutic genes. We examined whether eQTLs and PD‐associated GWAS signals share a common causal variant using the coloc.abf algorithm, which estimates posterior probabilities for five mutually exclusive hypotheses (PPH0–PPH4) (Lin et al. 2023). Default priors were: *P*_1_ = 1.0 × 10^−4^ for trait 1, *P*_2_ = 1.0 × 10^−4^ for trait 2, and *P*_12_ = 1.0 × 10^−5^ for both. The hypotheses were defined as: PPH0: No association for either trait. PPH1: Association with PD only. PPH2: Association with gene expression only. PPH3: Associations with both traits due to distinct causal variants. PPH4: Associations with both traits due to a shared causal variant. A PPH4 >75% was considered strong evidence of colocalization (Sun et al. 2024).

### 2.7 PheWAS

To systematically assess pleiotropy and potential adverse effects of candidate drug targets, we performed a PheWAS using the AstraZeneca PheWAS Portal (https://azphewas.com/) (Cao et al. 2023). This platform is based on whole‐exome sequencing data from the UK Biobank, which includes 281,104 European ancestry participants. A total of 2,108,983 genetic variants were tested for associations with 18,780 clinical phenotypes.

### 2.8 Identification of Therapeutic Targets via Knockout Mouse Models

To investigate the functional roles of specific genes in PD, we screened gene knockout (KO) mouse models using the mouse genome informatics (MGI) database (https://www.informatics.jax.org/). MGI is a comprehensive resource for genetic, genomic, and biological data in laboratory mice (Blake et al. 2021). KO mouse models are valuable tools for studying gene function, and phenotypic changes resulting from gene deletion provide essential insights into genes implicated in PD pathogenesis (Eppig et al. 2015).

### 2.9 Drug Prediction for Candidate Targets

To explore potential therapeutic interventions, we used the DSigDB database (https://dsigdb.tanlab.org/DSigDBv1.0/) to analyze drug gene interactions for the identified targets (Yoo et al. 2015). DSigDB is a resource linking drugs and other small molecules to their associated gene targets. We submitted the list of candidate genes to the platform and predicted drug candidates based on known or inferred gene–compound associations, assessing their potential for PD treatment.

## 3 Results

### 3.1 Therapeutic Genome

**FIGURE 2:**
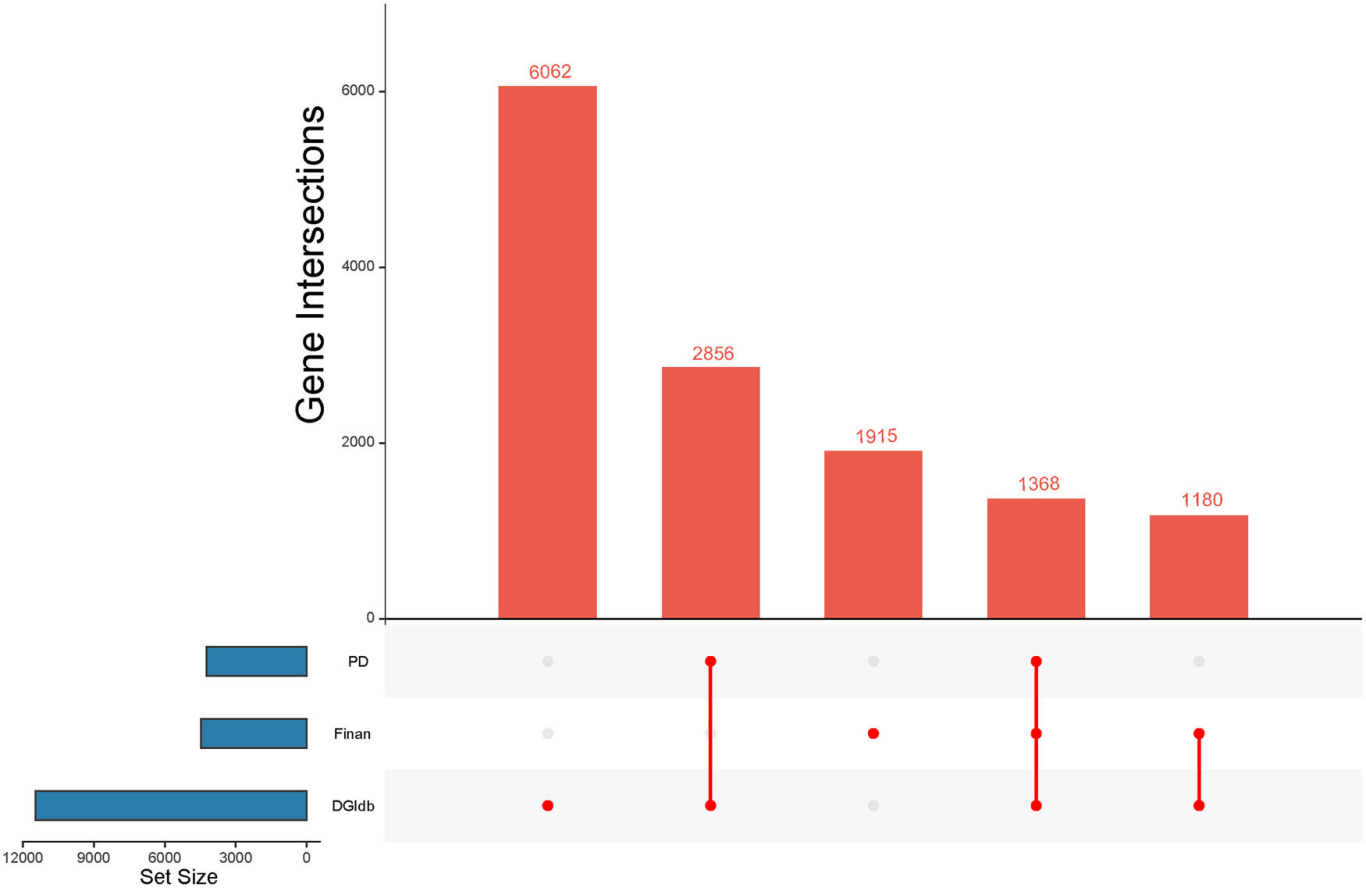
Visualization of the intersection relationships of therapeutic genes in PD.

A total of 11,466 candidate drug target genes were extracted from the DGIdb database. We then integrated data from existing literature to identify genes with experimentally validated druggability. Using the GeneCards database, we selected 4424 candidate genes strongly associated with PD, based on an integrated relevance score threshold of ≥1. Ultimately, 1368 highly associated drug target genes were identified. We designed and created the visualization shown in Figure 2, which presents the relationships between DGIdb, the Finan database, and PD‐related gene sets from a dual perspective—using both matrix combinations and bar plots. The figure illustrates the distribution of unique genes across various intersections.

### 3.2 Genome‐Wide Meta‐Analysis Identifies PD‐Associated Loci

**FIGURE 3:**
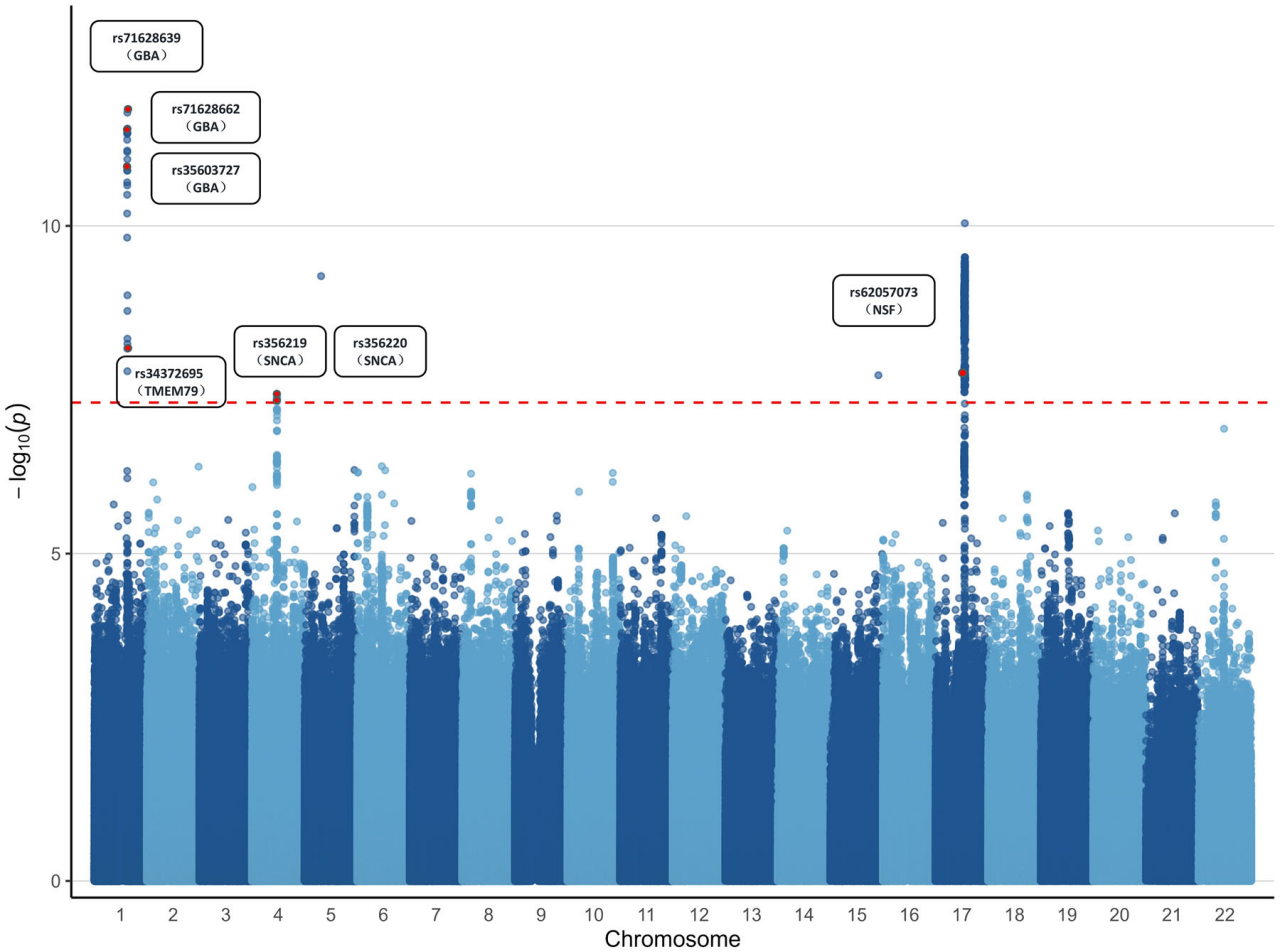
Genetic loci associated with PD identified through a genome‐wide association meta‐analysis integrating two studies.

A genome‐wide meta‐analysis was conducted using GWAS summary statistics from IPDGC and the Finnish Parkinson's Disease dataset. This analysis identified variants that were significantly associated with PD at the genome‐wide level (Figure 3). Specifically, we combined data from two studies: the IPDGC cohort (56,306 cases and 1,417,791 controls) and the Finnish PD dataset (4235 cases and 373,042 controls). After quality control, associations between 19,626,998 genetic variants and PD were analyzed. The analysis revealed 21 loci that reached genome‐wide significance for PD association (*p* < 5 × 10^−6^). The significant loci were annotated, and gene ontology (GO) enrichment analyses and Kyoto Encyclopedia of Genes and Genomes (KEGG) pathway enrichment analysis were performed. GO analysis indicated that the significant PD loci were associated with various biological processes (BP), including positive regulation of lymphocyte activation, interleukin‐mediated leukocyte adhesion, and T cell activation. Regarding cellular components (CC), the associated functions primarily involved endocytic vesicles, MHC protein complexes, and lysosomal membranes. For molecular functions (MF), key terms included MHC class II receptor activity, immune receptor activity, and ephrin receptor binding. Additionally, KEGG pathway enrichment revealed involvement in antigen processing and presentation, as well as Th1, Th2, and Th17 cell differentiation.

### 3.3 Eight Therapeutic Genes Significantly Associated With PD Risk

**FIGURE 4:**
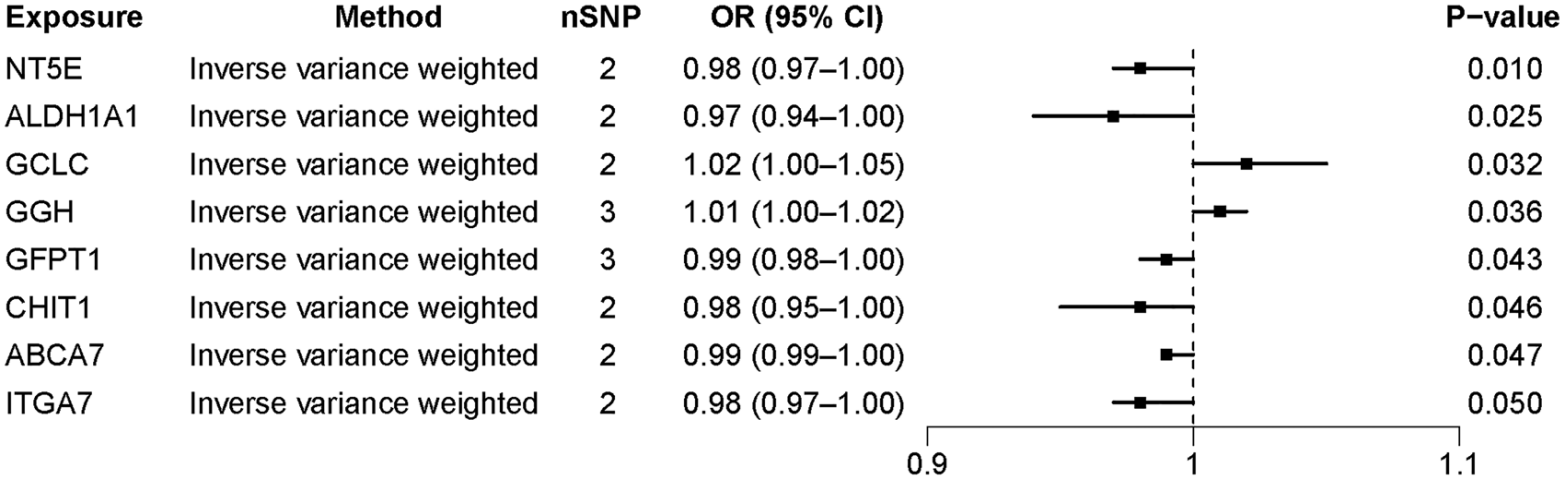
Forest plot showing significant associations between 8 therapeutic genes and PD risk.

We evaluated the causal effects of 1368 therapeutic genes on PD risk. Eight genes exhibited significant associations with PD risk (Figure 4). Elevated NT5E expression correlated with a lower PD risk (OR = 0.98, *P*  = 0.010). Increased ALDH1A1 expression exhibited a protective effect (OR = 0.97, *P*  = 0.025). Elevated GCLC expression heightened PD risk (OR = 1.02, *P*  = 0.032). Higher GGH expression was positively associated with PD risk (OR = 1.01, *P*  = 0.036). Higher GFPT1 expression was potentially linked to a reduced PD risk (OR = 0.99, *P * = 0.043). Expression changes in CHIT1 (OR = 0.98, *P*  = 0.046), ABCA7 (OR = 0.99, *P*  = 0.047), and ITGA7 (OR = 0.98, *P*  = 0.050) were likewise linked to PD risk.

### 3.4 Colocalization Analysis

A colocalization analysis was conducted to investigate whether PD and SNPs associated with therapeutic genes share a common causal genetic foundation. Of the eight candidate genes analyzed, five genes showed a high likelihood of harboring shared causal variants that affect both gene expression and PD risk (Table 1). Specifically, GCLC showed a PPH4 value of 0.78 at rs661603. ABCA7 had a PPH4 of 0.99 at rs113337161; ITGA7, 0.94 at rs73119219; GGH, 0.99 at rs61518338; and GFPT1, 0.99 at rs4854476. In contrast, no colocalization evidence was found for PD and the genes CHIT1, NT5E, or ALDH1A1. We identified GCLC, ABCA7, ITGA7, GGH, and GFPT1 as potential therapeutic targets, based on evidence indicating shared genetic influences between their expression quantitative trait loci (eQTLs) and the risk of PD.

**TABLE 1:** Colocalization results between SNPs of therapeutic genes and PD.

Gene	SNP	Dataset	Z‐score	r^2^	lABF	PPH4
GCLC	rs661603	df1	−17.88	0.997	156.47	0.78
		df2	1.56	0.999	−2.86	
ABCA7	rs113337161	df1	−28.94	0.986	410.7	0.99
		df2	1.35	0.999	−3.34	
ITGA7	rs73119219	df1	10.62	0.966	52.76	0.94
		df2	−1.78	0.999	−2.28	
GGH	rs61518338	df1	22.18	0.992	241.68	0.99
		df2	1.69	0.999	−2.53	
GFPT1	rs4854476	df1	37.55	0.997	699.99	0.99
		df2	1.82	0.995	−2.18	

### 3.5 PheWAS

To further evaluate whether the potential drug targets identified through colocalization analysis—GCLC, ABCA7, ITGA7, GGH, and GFPT1—exert beneficial or adverse effects on additional traits, we conducted a gene level PheWAS using data from the AstraZeneca PheWAS database (Table 2). The results were interpreted as associations between genetically regulated protein expression and specific diseases or traits. GCLC and GFPT1 were not significantly associated with other traits at the gene level (genome‐wide significance threshold, *P* < 5 × 10^−8^), unlike ABCA7, ITGA7, and GGH. These findings suggest that drugs targeting GCLC and GFPT1 may carry a lower risk of adverse effects and exhibit minimal horizontal pleiotropy.

**TABLE 2:** Traits significantly associated with ABCA7, ITGA7, and GGH identified through the AstraZeneca PheWAS portal.

Gene	Phenotype	Collapsing model	*p* value	No. samples	Effect size
ABCA7	Mean sphered cell volume	Ptv5pcnt	6.92 × 10^−12^	435,686	0.09
ITGA7	Forced expiratory volume in 1‐s	Ptv	5.11 × 10^−9^	2342	−4.23
ITGA7	Forced expiratory volume in 1‐s	Ptv5pcnt	5.11 × 10^−9^	2342	−4.23
ITGA7	Forced vital capacity	Ptv	8.33 × 10^−9^	2342	−4.2
ITGA7	Forced vital capacity	Ptv5pcnt	8.33 × 10^−9^	2342	−4.2
GGH	Cardiometabolic	Flexdmg	1.90 × 10^−15^	45,744	−1.38
GGH	Cardiometabolic	Ptvraredmg	4.14 × 10^−14^	45,744	−1.41
GGH	Cardiometabolic	Raredmg	2.97 × 10^−11^	45,744	−1.28
GGH	Cardiometabolic	UR	3.20 × 10^−9^	45,744	−1.76

### 3.6 Identification of Mouse Knockout Models for Therapeutic Genes

To investigate whether therapeutic genes can induce PD‐related phenotypic changes, we systematically analyzed various KO mouse models listed in MGI database (see Table S1). The results indicated that the GCLC and GFPT1 knockout models exhibited significant neurological abnormalities, including: 1) disorganization of the cingulate region, accompanied by noticeable abnormalities in myelin morphology; 2) a marked reduction in myelin thickness compared to the control group, suggesting impaired myelin development; 3) abnormal arrangement of neuromuscular junction structures, which may affect signal transmission efficiency; and 4) a significant increase in serum levels of alanine aminotransferase (ALT) and aspartate aminotransferase (AST).

### 3.7 Candidate Drug Prediction

This study utilized the DSigDB database to predict potential agents. Eight candidate compounds were identified based on adjusted *P*‐values (Table 3). Paraquat and Maneb were significantly associated with GCLC, with adjusted *P*‐values of 0.029 and 0.018. In drug‐induced PD, Spiperone showed significant interactions with both GCLC and GFPT1 (adjusted *p* = 0.018). Chlorpromazine and Thioridazine also demonstrated close associations with GFPT1, with adjusted *P*‐values of 0.032 and 0.049. In the dopaminergic system, Dopamine exhibited a strong enrichment in its interaction with GCLC (adjusted *p* = 0.031). Among antioxidants, Glutathione and Ebselen exhibited significant associations with GCLC, with adjusted *P*‐values of 0.034 and 0.023.

**TABLE 3:** Candidate drugs predicted using the DSigDB database.

Category	Compound	Fold enrichment	p.adjust	GeneID
Environmental toxins	Paraquat	46.12	0.029	GCLC
	Maneb	316.89	0.018	GCLC
Drug‐induced Parkinsonism	Spiperone	56.62	0.018	GCLC/GFPT1
	Chlorpromazine	37.07	0.032	GFPT1
	Thioridazine	20.64	0.049	GFPT1
Dopaminergic system	Dopamine	39.77	0.031	GCLC
Antioxidant	Glutathione	34.35	0.034	GCLC
	Ebselen	72.23	0.023	GCLC

## 4 Discussion

This study conducted a genome‐wide meta‐analysis and identified 21 genetic variants significantly associated with PD. Subsequent gene annotation and pathway enrichment analyses revealed that these variants participate in biological processes such as lymphocyte regulation, leukocyte adhesion, and T‐cell activation. These variants are also associated with cellular components such as endocytic vesicles and MHC complexes, as well as molecular functions such as MHC class II receptors. Previous GWAS have identified a link between PD and specific MHC class II gene haplotypes, revealing multiple polymorphic sites in non‐coding regions that regulate MHC II expression (Garretti et al. 2019). In the brains of patients with PD, neuroinflammation is marked by microglial activation, elevated MHC II expression, increased levels of inflammatory mediators, and T lymphocyte infiltration (Jimenez‐Ferrer et al. 2021). Pro‐inflammatory Th1 and Th17 cells amplify inflammation by releasing cytokines. Th2 cells and regulatory T cells regulate immune responses by modulating B cell activity and suppressing inflammation. Compared with healthy controls, patients with PD show a higher proportion of Th1 cells and a slightly reduced proportion of Th2 cells in peripheral blood (Liu et al. 2022). In addition to PD, similar immune dysregulation patterns are observed in other neurodegenerative disorders. In Alzheimer's disease, abnormal expression of MHC class II molecules is frequently observed (Altendorfer et al. 2025), accompanied by dysregulated activation of Th1 and Th2 helper T cell subsets (Jafarzadeh et al. 2023). In both animal models and brain tissues of amyotrophic lateral sclerosis patients, T cell dysregulation and hyperactivation of glial cells have been reported (Jin et al. 2020). By integrating data from DGIdb, GeneCards, and Finan databases, we identified 1,368 therapeutic genes strongly associated with PD. Using MR, colocalization analysis, and PheWAS, we identified genetic loci associated with PD and pinpointed therapeutic genes significantly related to PD risk.

To validate candidate therapeutic genes, we evaluated their causal relationship with PD risk. The analysis revealed strong associations between GCLC, GFPT1, NT5E, ALDH1A1, and PD risk. Colocalization analysis identified shared causal variants between gene expression of NT5E, ALDH1A1, GCLC, GGH, GFPT1, CHIT1, ABCA7, ITGA7, and PD risk, supporting their potential as therapeutic targets. PheWAS analysis further showed that GCLC and GFPT1 had fewer pleiotropic associations with other clinical traits, suggesting lower risk of off‐target effects. KO models in mice revealed significant neurological abnormalities upon the knockout of GCLC and GFPT1, including structural disorganization in the cingulate cortex, impaired myelination, and structural abnormalities in neuromuscular junctions. The knockout mice also showed significantly elevated levels of ALT and AST. Analysis of the DSigDB database identified eight potential compounds targeting GCLC and GFPT1, including Paraquat, Spiperone, and Glutathione. Several compounds, including dopamine and glutathione, have demonstrated neuroprotective mechanisms in neurodegenerative diseases (Latif et al. 2021; Bjørklund et al. 2021). Glutathione (GSH), a ubiquitous thiol containing tripeptide, mitigates oxidative stress induced cellular damage by scavenging reactive oxygen species (ROS) (Diaz‐Vivancos et al. 2015). PD models typically exhibit elevated oxidative stress and reduced GSH levels (Chang and Chen 2020). GSH synthesis relies on the intermediate γ‐glutamylcysteine (γ‐GC), catalyzed by the rate‐limiting enzyme glutamate‐cysteine ligase (GCL) (Niu et al. 2025). GCL consists of two subunits: the catalytic GCLC and the regulatory GCLM. Animal studies show that in the MPTP‐induced PD model, the level of the oxidative stress marker TBARS is elevated, and the expression of the antioxidant enzyme GCLC is increased (Lu et al. 2014). Studies have associated GCLC gene polymorphisms with several diseases, including stroke (Campolo et al. 2007), reduced lung function (Siedlinski et al. 2008), psoriasis (Efanova et al. 2023), and preeclampsia (Li et al. 2020). GFPT1 is involved in the synthesis of UDP‐*N*‐acetylglucosamine (UDP‐GlcNAc), a key substrate for protein O‐GlcNAc modification (Farshadyeganeh et al. 2023). Elevated O‐GlcNAc levels have been shown to exert neuroprotective effects and delay neurodegeneration (Kim et al. 2024).

This study enhances our understanding of the genetic mechanisms underlying PD and identifies potential therapeutic targets. However, several limitations persist. First, the GWAS data are largely derived from individuals of European ancestry. Although this enhances statistical power due to large sample sizes, it may limit the generalizability of the findings to non‐European populations. Second, the quantitative trait locus (QTL) data utilized in this study are mainly derived from peripheral blood samples. Although suitable for large‐scale analyses, peripheral blood may not accurately represent gene expression in brain regions relevant to PD. Third, although GCLC and GFPT1 knockout mouse models have demonstrated their roles in the nervous system, systemic knockout approaches may lead to nonspecific phenotypes or compensatory developmental responses, limiting their ability to fully model the complex pathology of human PD. Finally, the current analysis primarily focuses on causal inference at the single‐gene level, with limited consideration of genetic interactions. Given that PD is a typical polygenic complex disease, it is recommended to integrate gene co‐expression networks, protein‐protein interaction maps, and pathway enrichment analyses.

## 5 Conclusion

In conclusion, this study integrated large‐scale population genetic data with causal inference, colocalization analysis, mouse model validation, and drug prediction to systematically evaluate the roles of GCLC and GFPT1 in PD. These genes were identified as novel therapeutic targets with promising translational potential.

## Author Contributions

**Shan Yang**: conceptualization, investigation, methodology, visualization, writing – review and editing, software, writing – original draft. **Qinfen Wu**: data curation, software, methodology, conceptualization. **Xinling Yang**: writing – review and editing, resources, supervision

## Funding

This study was supported by the Xinjiang Uygur Autonomous Region Key Research and Development Program (2023B03003), the Young Top Talent Project of Xinjiang Uygur Autonomous Region (2023TSYCCX0071), and the Graduate Innovation Program of Xinjiang Uygur Autonomous Region (XJ2025G188).

## Ethics Statement

Ethical approval was not necessary for this study because it did not involve any direct participation of individual patients at any stage. The analysis was exclusively based on publicly available GWAS data.

## Conflicts of Interest

The authors declare no conflicts of interest.

## Supporting information




**Supplementary Table**: brb371087‐supp‐0001‐TableS1.xlsx

## Data Availability

Data sharing not applicable to this article as no datasets were generated or analyzed during the current study.
